# Ecological responses of Antarctic *Chaetoceros* spp. to simulated melting and salinity shifts

**DOI:** 10.3389/fmicb.2026.1750888

**Published:** 2026-02-19

**Authors:** Savannah Zigic, Olga Mangoni, Emanuela Serino, Francesco Bolinesi

**Affiliations:** 1 Department of Biology, University of Naples Federico II, Naples, Italy; 2 Consorzio Nazionale Interuniversitario per le Scienze del Mare (CoNISMa), Rome, Italy

**Keywords:** Antarctic phytoplankton, climate change, functional traits, HPLC, Ross Sea

## Abstract

Climate-driven freshening and shifting sea-ice dynamics are altering surface salinity regimes in coastal Antarctic waters, with profound ecological consequences. Phytoplankton, as the foundation of polar marine food webs and a key driver of biogeochemical cycles, are particularly sensitive to salinity variability. In this study, we exposed a mixed *Chaetoceros* culture from the Terra Nova Bay (the Ross Sea) to a range of salinity conditions representative of both recent microhabitats and projected future scenarios. By tracking short-term and acclimated responses across multiple functional traits—including photosynthetic efficiency, cell size and morphology, pigment composition, and nutrient uptake—we identified distinct acclimation strategies shaped by the severity and direction of salinity stress. These findings reveal how salinity fluctuations can restructure phytoplankton physiology in ways that influence trophic transfer efficiency, carbon export potential, and community resilience. For instance, shifts toward smaller, less-pigmented cells under moderate hyposalinity reduce food quality for grazers and alter energy flow through the food web, while extreme salinity events favor microbial recycling over carbon export. Moreover, salinity-driven changes in pigment:chlorophyll a ratios have implications for interpreting remote sensing data and chemotaxonomic reconstructions. By linking physiological plasticity to ecosystem-level processes, this study underscores the central role of salinity as an ecological filter in polar systems and highlights the need to incorporate salinity variability into models of phytoplankton dynamics and Southern Ocean biogeochemistry under climate change.

## 1 Introduction

Marine ecosystems of coastal Antarctica are shaped by the interplay of sea ice dynamics, seasonal light availability, and vertical mixing processes, which together generate spatially heterogeneous and temporally variable habitats for phytoplankton (Bolinesi et al., 2020b; Mangoni et al., 2004, 2017; Moro et al., 2000; Saggiomo et al., 2017, 2021a,b; Massi et al., 2025). In polynya systems such as Terra Nova Bay (TNB, the Ross Sea), strong katabatic winds, and persistent sea ice formation drive brine rejection, leading to the production of high-salinity shelf waters (Bromwich and Kurtz, 1984; Hollands and Dierking, 2016). Conversely, seasonal warming and ice melt inject pulses of low-salinity meltwater into the upper mixed layer. The opposing processes—ice formation and melting—create a highly dynamic and heterogeneous environment. Furthermore, these processes give rise to a wide range of physical and chemical conditions, including the development of brine channels within developing sea ice, the release of freshwater during melt events, and the extrusion of concentrated saline brines during ice growth (Testón-Martínez et al., 2024). The interplay between freezing and melting not only modulates stratification and circulation but also influences biogeochemical fluxes and ecosystem structure across the polynya. Apart from this, these processes also give rise to a mosaic of co-occurring microenvironments—including hypersaline brine channels and pockets embedded within sea ice, the under-ice boundary layer, and surface strata influenced by seasonal meltwater inputs—each defined by steep and dynamic gradients in salinity, temperature, light availability, and nutrient concentrations (Eicken, 2003; Fraser et al., 2023).

The physiological responses and taxonomic composition of phytoplankton communities are highly sensitive to various variables, often exhibiting non-linear and threshold-dependent dynamics (Arrigo et al., 2010; Hoppe et al., 2018; Boyd et al., 2016; Petrou et al., 2016; Hayward et al., 2025). Consequently, spatial and temporal variability in salinity emerges as a key regulator of phytoplankton succession, bloom initiation and termination, and the modulation of biogeochemical fluxes in coastal Antarctic marine systems (Arrigo and McClain, 1994; Arrigo et al., 1998, 1999; Smith et al., 2014).

Diatoms are the principal contributors to primary production in many Antarctic coastal ecosystems, where they play a pivotal role in driving particulate organic carbon (POC) export, regulating silica cycling, and shaping the nutritional quality of food available to higher trophic levels (Arrigo et al., 2008; Smith et al., 2014). Among Antarctic diatoms, the centric genus *Chaetoceros* is particularly widespread and ecologically influential, dominating both pelagic and sympagic communities in regions such as the Ross Sea (Crosta et al., 1997; Moro et al., 2000; Gogorev and Samsonov, 2016).

*Chaetoceros* species are well adapted to the extreme variability of polar environments: they colonize brine channels and platelet ice during winter and early spring, act as seed populations for under-ice and surface blooms, and form extensive pelagic blooms under shallow mixed layers and high irradiance conditions (Crosta et al., 1997; Moro et al., 2000; Ligowski et al., 2012; Gogorev and Samsonov, 2016; Saggiomo et al., 2021b; Stuart et al., 2025). Their ecological success is underpinned by a suite of physiological traits, including dynamic regulation of photoprotective pigments, formation of resting spores, synthesis of osmolytes and antifreeze proteins, and plasticity in nutrient allocation strategies (French and Hargraves, 1985; Crosta et al., 1997; Plettner, 2002; Gwak et al., 2010; Van de Poll et al., 2011; Kim et al., 2017).

However, the relative contribution of intraspecific vs. interspecific variability to the physiological responses of Antarctic *Chaetoceros* remains insufficiently resolved. Empirical data on short-term vs. acclimated responses in Antarctic *Chaetoceros* taxa are still limited, and different strains within the same species exhibit markedly different tolerances to abrupt salinity shifts. Mixed cultures used in this study may therefore capture a broader and more ecologically realistic range of strain-level responses than single-strain isolates.

Climate forcing is altering the frequency and magnitude of salinity regimes experienced by coastal phytoplankton. In the Ross Sea, episodic reductions in sea ice extent, increased meltwater inputs, and circulation changes have driven regional freshening trends in recent decades (Castagno et al., 2019; Jacobs et al., 2022; Jena et al., 2024), while ice formation and melt processes still produce transient hypersaline plumes (Eicken, 2003; Widell et al., 2006; Peterson, 2018; Porter et al., 2019; Fraser et al., 2023; Miller et al., 2024). Consequently, planktonic populations may experience abrupt salinity spikes or dips lasting seconds to minutes during brine drainage or sustained hypo−/hypersaline periods that persist for days to weeks in stratified layers or isolated brine pockets. Distinguishing physiological outcomes produced by stress severity (moderate vs. extreme), exposure duration, and change direction (hypo vs. hypersalinity) is required to predict phytoplankton responses across future scenarios.

Prior experimental works on salinity impacts have emphasized temperate species or combined stressors; few studies isolate abrupt salinity changes in Antarctic diatoms. Existing polar studies indicate osmoregulation, antioxidant activation, and xanthophyll cycle engagement under stress, but these studies often address gradual acclimation or combined drivers (Gleitz and Thomas, 1992; Petrou et al., 2011; Hernando et al., 2015, 2018; Bozzato et al., 2019; Antoni et al., 2020). Important but unresolved questions include: (1) What are the immediate (hours) physiological responses to an abrupt salinity perturbation akin to brine plumes? (2) Can Antarctic *Chaetoceros* acclimate during days to weeks after the salinity shift, and what trade-offs arise between population growth, cell size, and pigment composition? (3) Do stress severity (moderate vs. extreme) and direction (hypo vs. hypersalinity) differentially structure responses? and (4) How does salinity modulate pigment: Chl a ratios applied in chemotaxonomy?

We addressed these questions by exposing a mixed *Chaetoceros* culture (Terra Nova Bay isolate) to a salinity gradient representative of present-day microenvironments and potential future freshening (20‰, 24‰, 34‰, 44‰, and 60‰). We tracked immediate (2 h) and longer-term (4, 8, 12, and 15 days) responses across multiple functional endpoints—maximum quantum efficiency (Fv/Fm), cell density and growth dynamics, cell size and morphology, chlorophyll a content (total and per cell), high-performance liquid chromatography (HPLC) pigment profiles and xanthophyll activity, nitrate and phosphate uptake, and N:P ratios. Our objectives include (1) characterizing short-term vs. acclimation responses to abrupt salinity change, (2) evaluating whether stress severity or direction of salinity change (increase vs. decrease) primarily structures physiological trajectories, (3) identifying trade-offs between growth, size, and pigment investment with ecological consequences, and (4) providing salinity-specific pigment:Chl a reference data for CHEMTAX applications. By combining high-resolution temporal sampling with multiple functional endpoints, this study clarifies mechanistic pathways through which salinity variability may reconfigure phytoplankton performance and community outcomes in a changing Southern Ocean.

## 2 Materials and methods

### 2.1 Culture origin and maintenance

*Chaetoceros* spp. were isolated from surface water (10 m) collected from Terra Nova Bay (Ross Sea) during the P-ROSE cruise (R/V Italica, January 2017; 74.7557°S, 164.1775°E). The mixed-species culture was maintained at 34‰ in L1 + Si medium (Guillard and Hargraves, 1993) at 4 °C under low irradiance (5 μmol photons m^−2^ s^−1^, cool white LEDs). The light level reflects the lower range of irradiance conditions typical of the study area. The time between field isolation and the experiment was approximately 36 months, during which cultures were maintained in the exponential phase to preserve physiological relevance.

### 2.2 Experimental design and treatments

The salinity treatments were selected to encompass the osmotic range observed in Antarctic coastal and under-ice environments, where strong hyposaline and hypersaline conditions arise from meltwater inputs, sea ice brine rejection, and polynya dynamics. Accordingly, five treatments were established spanning from extreme hyposaline to extreme hypersaline conditions: 20‰ (extreme hyposalinity), 24‰ (moderate hyposalinity), 34‰ (control), 44‰ (moderate hypersalinity), and 60‰ (extreme hypersalinity). Media were prepared by mixing 38‰ filtered natural seawater, Milli-Q water, and reagent-grade NaCl and were enriched with L1 nutrients and silicate immediately before inoculation. For each salinity-time point (day 0.1: 2 h, day 4, day 8, day 12, and day 15), three biological replicates were incubated in 250 mL sterile glass flasks (75 flasks total). Incubation conditions matched culture maintenance at 4.0 °C, 5.0 μmol photons m^−2^ s^−1^, and gentle stirring/rocking to avoid settling.

### 2.3 Photo-physiology (Fv/Fm)

Maximum quantum efficiency of PSII (Fv/Fm) was measured with a PHYTO-PAM (Walz) using Phyto-Win software. Samples (10 mL) were dark-adapted for ≥30 min prior to measurement, which was conducted in triplicate. Baseline blanks matched sample salinity, and instrument gain was adjusted per run. However, Fv/Fm was calculated as (Fm−F0)/Fm from three consecutive saturated pulses.

### 2.4 Cell density, size, and morphology

**Figure 1:**
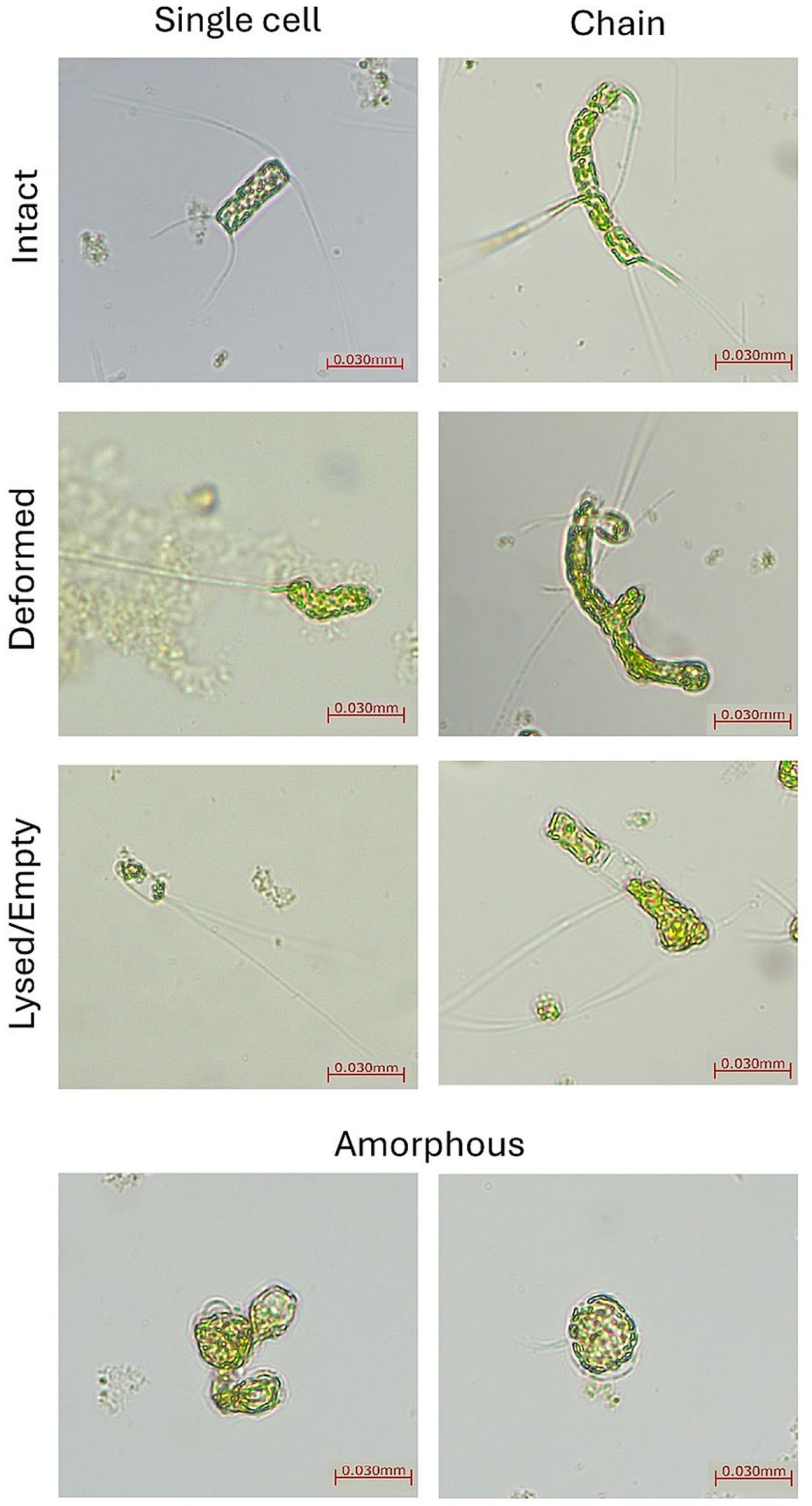
Representative light microscopy images (20×) of *Chaetoceros* cells classified into seven morphological categories: intact single, deformed single, lysed/empty single, intact chain, deformed chain, lysed/empty chain, and amorphous.

Samples were fixed with Lugol’s iodine, and cell densities were estimated using Utermöhl sedimentation and inverted microscopy (Leica DMIL LED). Sedimentation chambers used a 1:1 fixed sample:media ratio; counts were performed in multiple fields and averaged. Density calculations were corrected for dilution factors (Zigone et al., 2010). For morphometry, images were acquired at 20 × magnification with a Leica 540/0.45 camera and analyzed with ImageJ. Only intact single cells in the girdle view were measured along the apical axis (excluding setae). Chains (≥2 attached cells) were counted by cell number but measured as aggregates where needed. Each replicate included 100 measured cells (*n* = 300 per salinity × time) for size and categorical morphology classification into seven classes: intact single, deformed single, lysed/empty single, intact chain, deformed chain, lysed/empty chain, and amorphous (Figure 1).

### 2.5 Chlorophyll a and pigment analysis

Chlorophyll a was determined fluorometrically after acetone extraction (90% acetone; Neveux and Panouse, 1987 method) using a Shimadzu RF-6000. For pigment analysis, 100 mL aliquots were vacuum filtered onto 25 mm Whatman GFF filters, stored at −20 °C, and extracted with 90% methanol. HPLC separation followed Vidussi et al. (1996) using a reverse-phase Thermo Scientific MOS-2 HYPERSIL column and photodiode array detection at 440 nm. Pigment identification and quantification used calibration with 20 standards; pigment concentrations were normalized to Chl a to produce pigment:Chl a ratios. Xanthophyll cycle activity was expressed as percent de-epoxidation state (%DES = DT/(DD + DT) × 100).

### 2.6 Nutrients and pH

Nitrate (N-NO_3_^−^) and phosphate (P-PO_4_^3−^) were measured on 20 mL filtered samples (0.2 μm) using an EasyChem Plus discrete analyzer following established protocols—nitrate:vanadium reduction; phosphate:molybdate blue (Hansen and Grasshoff, 1983). Calibration standards were adjusted to the salinity of each treatment to avoid interferences in the reactions and measurements (i.e., for 20 and 24 PSU samples, standards were prepared at 22 PSU; for all others, standards matched the sample salinity of 34, 44, and 60 PSU). N:P ratios were calculated for each biological replicate using the measured nitrate and phosphate concentrations; the reported mean and standard deviations therefore reflect replicate-level variability rather than propagated analytical uncertainty. Media pH was measured at T15 with a Thermo Scientific Orion Star A11 pH meter, and parallelly, blanks (uninoculated media) were measured.

### 2.7 Statistical analyses

Summary statistics and plots were produced using R (v4.0.3) and PAST 5. Multivariate ordination was performed using principal component analysis (PCA) to assess covariation among ecological variables (Chl a, pheopigments, cell density, Chl a:Pheo ratio, Chl a per cell, Fv/Fm, N:P ratio, mean cell length, and fucoxanthin). PCA was conducted in PAST 5 on the variance–covariance matrix after standardizing all variables to a zero mean and unit variance (*z*-scores). The number of components retained was determined from the scree plot and the cumulative variance explained, with interpretation focused on PC1 (63.0%) and PC2 (24.9%). In the biplot, points represent individual samples projected into principal component space, while vectors indicate variable loadings. A two-way PERMANOVA (the Bray–Curtis index; permutations = 9,999) was used to test the effects of salinity, time, and their interaction on the multivariate trait matrix. *Post hoc* interpretation was based on ordination patterns and PERMANOVA outputs, supporting the identification of distinct acclimation strategies. Reported values are mean ± SD unless otherwise stated.

## 3 Results

### 3.1 Photosynthetic efficiency (Fv/Fm) and acclimation patterns

**Figure 2:**
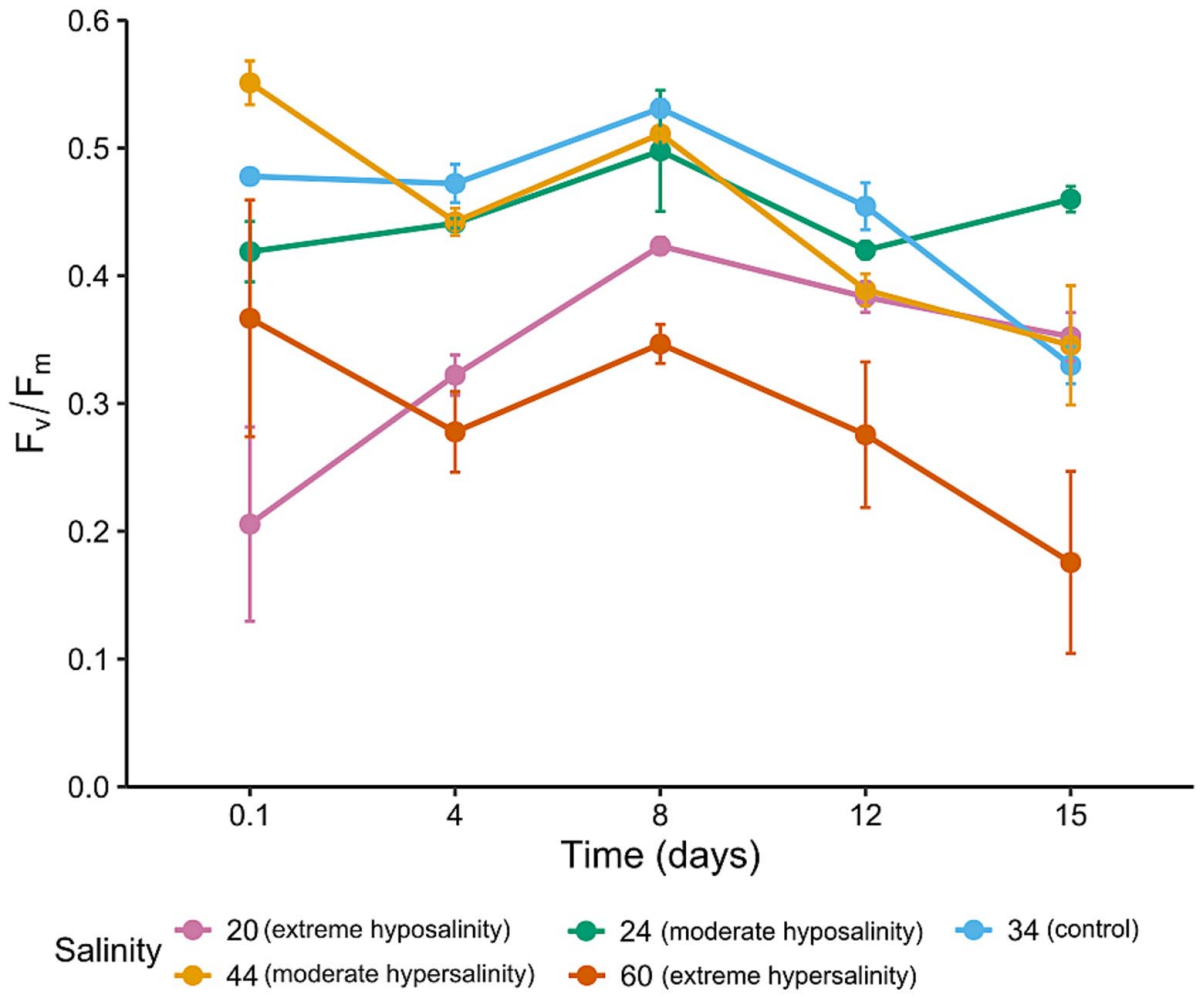
Maximum quantum efficiency (Fv/Fm) for *Chaetoceros* spp. across five salinity treatments (20‰, 24‰, 34‰, 44‰, and 60‰) measured on days 1 (2 h), 4, 8, 12, and 15. Points = mean ± SD (*n* = 3). Inset legend indicating salinities and time points.

Maximum quantum efficiency (Fv/Fm; Figure 2) varied significantly across salinity treatments and over time, revealing distinct acclimation trajectories. Cultures maintained at control salinity (34‰) exhibited high Fv/Fm values until day 8, indicating optimal photosynthetic performance and minimal stress. Moderate salinity deviations (24‰ and 44‰) elicited divergent responses: at 24‰, Fv/Fm declined within the first 2 h, suggesting acute stress, but recovered progressively by day 8, suggesting potential acclimation. In contrast, cultures at 44‰ showed a net increase within the first 2 h, followed by a slight decrease over time. Extreme salinity treatments (20‰ and 60‰) caused pronounced and persistent reductions in Fv/Fm. At 20‰, values dropped below 0.2 within hours, accompanied by visible signs of cell deformation and lysis. A small surviving fraction showed partial recovery by day 8, possibly reflecting selection for tolerant subpopulations. At 60‰, the Fv/Fm remained low throughout the experiment, with no significant recovery, indicating sustained photoinhibition and compromised photosynthetic capacity.

### 3.2 Cell density, growth dynamics, and chlorophyll-a allocation

**Figure 3:**
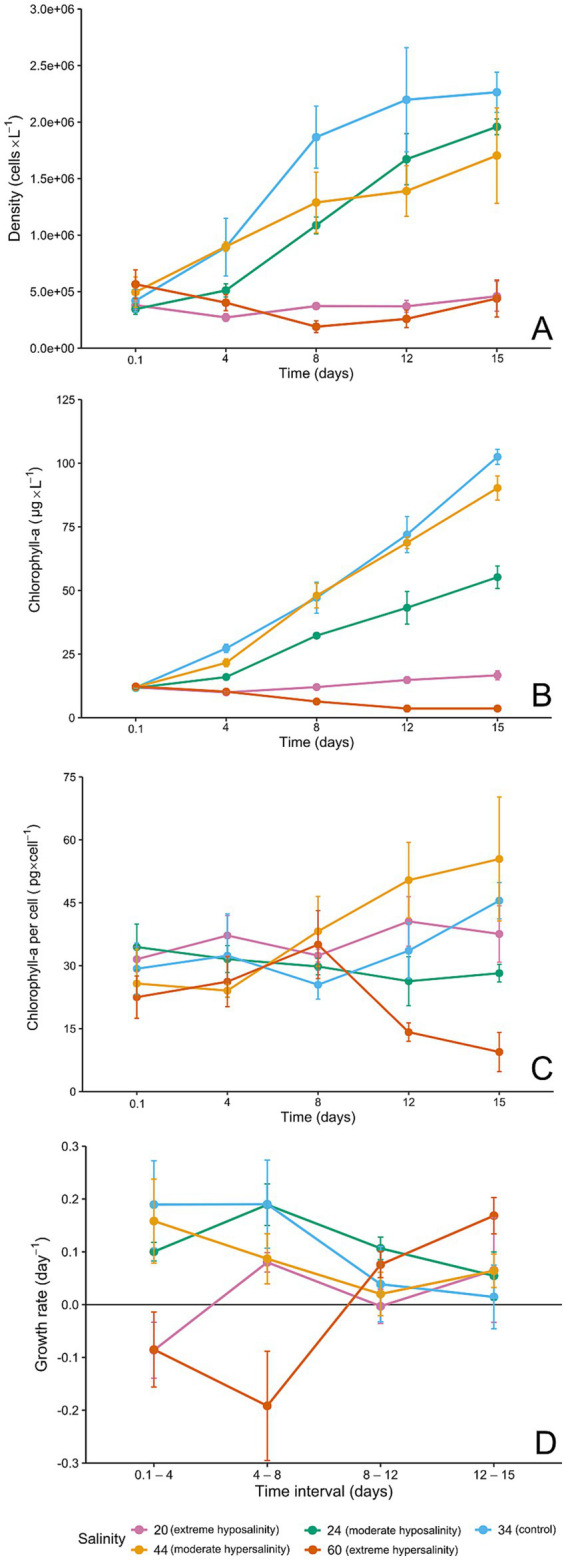
**(A)** Cell density (cells × L^−1^) through time for each salinity, **(B)** chlorophyll *a* concentration (μg × L^−1^) through time, **(C)** chlorophyll *a* per cell (pg × cell^−1^) through time, error bars = ± SD (*n* = 3), and **(D)** growth rate (day^−1^) in time intervals (days). Panels are arranged from top to bottom.

Population growth (Figure 3A) and biomass accumulation (Figure 3B) were strongly influenced by salinity. Control cultures exhibited consistent exponential growth, reaching peak cell densities by day 15. Moderate treatments (24‰ and 44‰) supported growth, but both showed reduced performance compared with the control and followed contrasting physiological adjustments. At 24‰, cultures exhibited reduced chlorophyll a content per cell (Figure 3C) and smaller cell sizes, together with a modest decline in growth rate, indicating physiological stress under moderate hyposalinity.

Conversely, cultures at 44‰ grew more slowly but maintained a higher concentration of chlorophyll a per cell, reflecting increased per-cell pigment investment under moderate hypersalinity. Extreme salinities (20‰ and 60‰) severely impaired population growth, and chlorophyll a pools declined progressively at 60‰ and remained relatively constant at 20‰, accompanied by signs of cellular lysis and deformation.

Growth rates (*μ*) calculated during the exponential phase revealed clear differences among salinity treatments (Figure 3D). Control cultures at 34‰ exhibited the highest growth rate (*μ* = 0.190 ± 0.047 d^−1^), followed by 24‰ (*μ* = 0.183 ± 0.039 d^−1^). Cultures at 44‰ showed a reduced growth rate (*μ* = 0.087 ± 0.047 d^−1^), while extreme salinities (20‰ and 60‰) exhibited substantially lower *μ* values (*μ* = 0.080 ± 0.018 d^−1^ and *μ* = 0.076 ± 0.025 d^−1^, respectively). The two-way ANOVA showed that salinity had a significant effect on growth rates (*F* = 10.61, *p* < 0.001), whereas the main effect of time interval was not significant (*p* = 0.58). However, we detected a strong salinity × time-interval interaction (*F* = 9.99, *p* < 0.001), indicating that the response of *μ* to salinity changed over the course of the incubation. To further explore this interaction, we performed one-way ANOVAs for each time interval. Significant differences among salinity treatments were observed during 0.1–4 h (*F* = 12.36, *p* < 0.001) and 4–8 h (*F* = 16.76, *p* < 0.001). In contrast, no significant differences were detected during 8–12 h (*p* = 0.06) and 12–15 h (*p* = 0.09). Tukey HSD *post hoc* tests revealed that, in the first two intervals, the growth rates at extreme salinities (20‰ and 60‰) were significantly lower than those at intermediate salinities (24–44‰). These results quantitatively support the observed pattern of reduced *μ* under both low and high salinity stress.

### 3.3 Morphological responses and cell size variation

**Figure 4:**
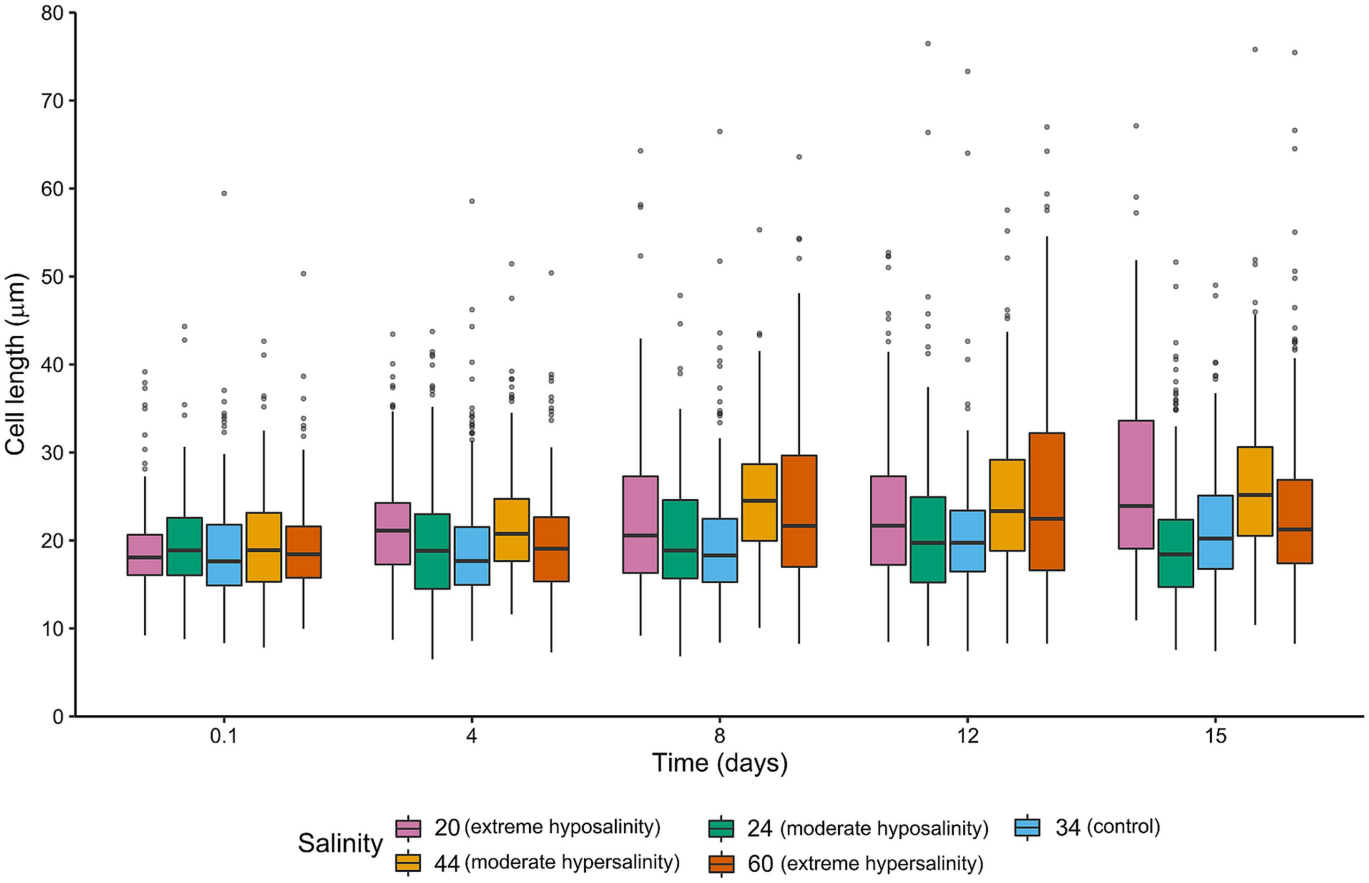
Boxplots of intact single cell length (μm) measured along the apical axis for each salinity and time point. Boxes indicate median and interquartile range; whiskers = 1.5 × IQR.

**Figure 5:**
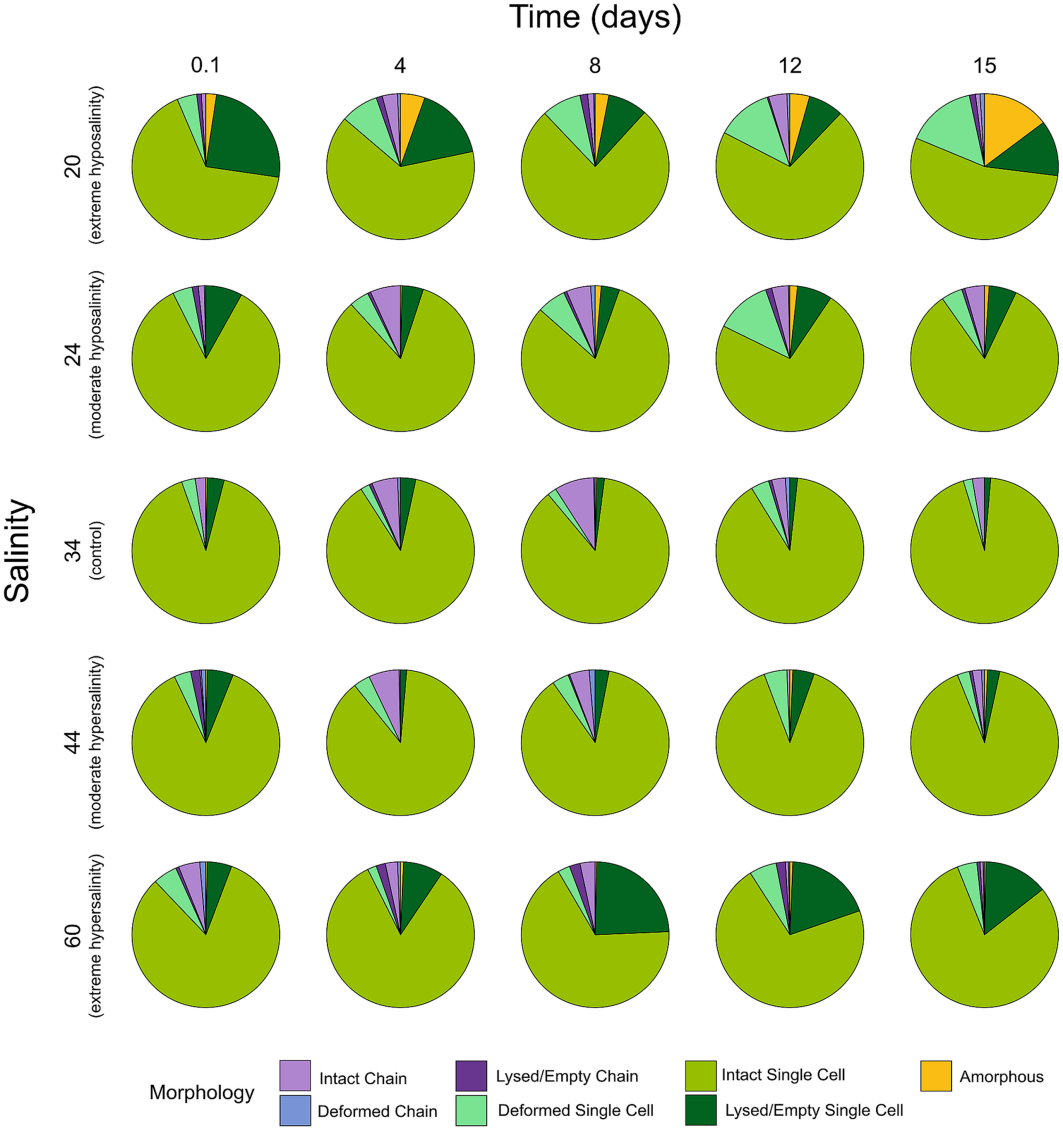
Proportional distribution of morphological categories (intact single, deformed single, lysed/empty single, intact chain, deformed chain, lysed/empty chain, complete lysis/amorphous) for each salinity across five sampling times (*n* = 300 cells per salinity × time).

**Figure 6:**
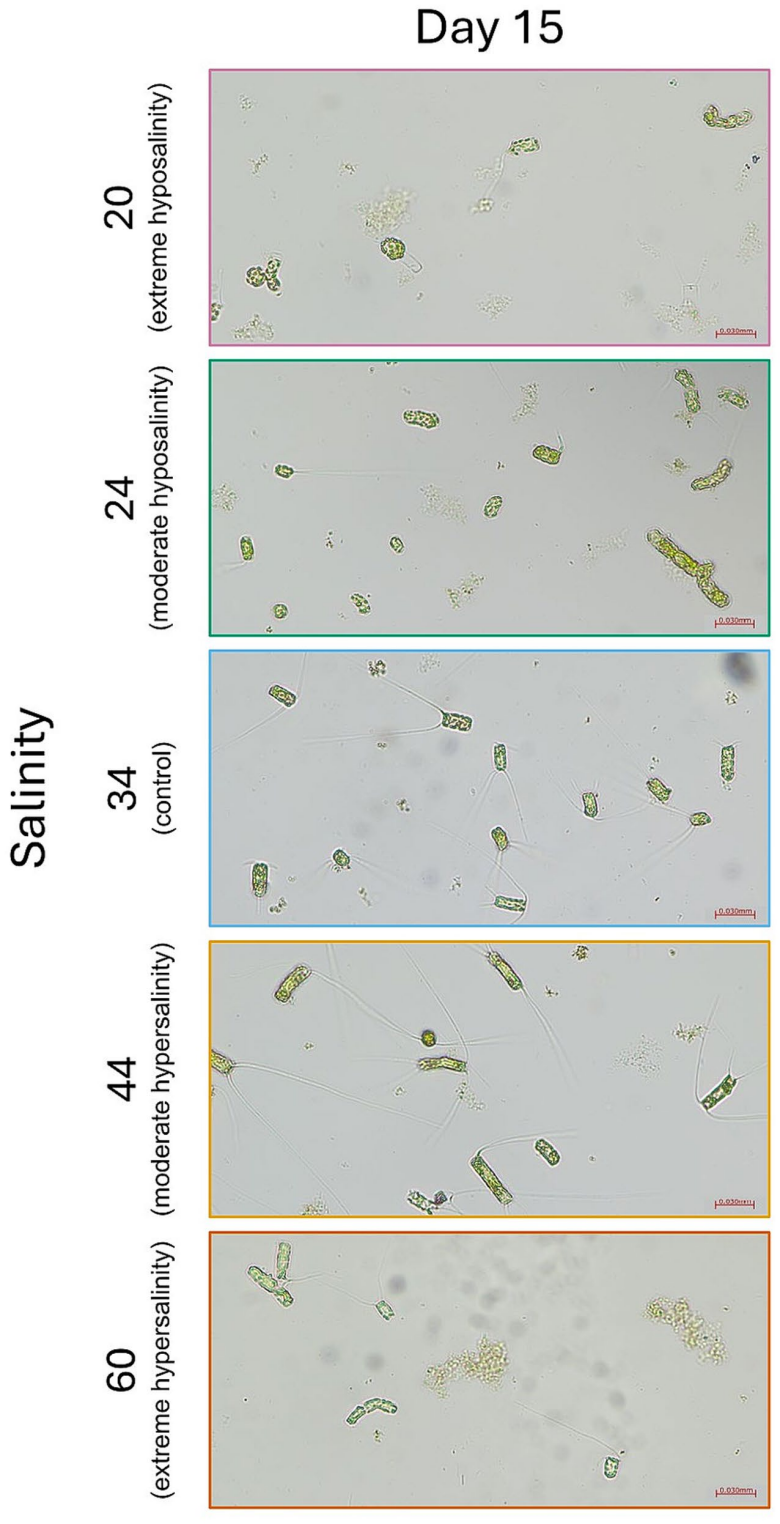
Representative light microscopy images (20×) of *Chaetoceros* cells fixed on day 15 for each salinity treatment (20‰, 24‰, 34‰, 44‰, 60‰). Scale bar = 30 μm. Panels are labeled by salinity.

Cell size (Figure 4) and morphology (Figures 5, 6) were markedly affected by salinity treatments. At 24‰, cells were markedly smaller and more rounded, consistent with accelerated division and reduced investment in structural integrity. A moderate increase in deformation, lysis, and amorphous morphologies was observed relative to the control, although these alterations were less pronounced than those recorded at 20‰. This morphological shift aligns with the observed increase in population density and reduced pigment per cell. At 44‰, cells were larger, more elongated, and exhibited thicker frustules, indicative of increased per-cell investment and a shift toward predation by larger grazers. Cultures at 20‰ showed widespread deformation, swelling, and lysis, with irregular cell shapes and compromised integrity. At 60‰, cells appeared shrunken and fragmented, with signs of cytoplasmic collapse.

### 3.4 Pigment composition and photoprotective strategies

**Figure 7:**
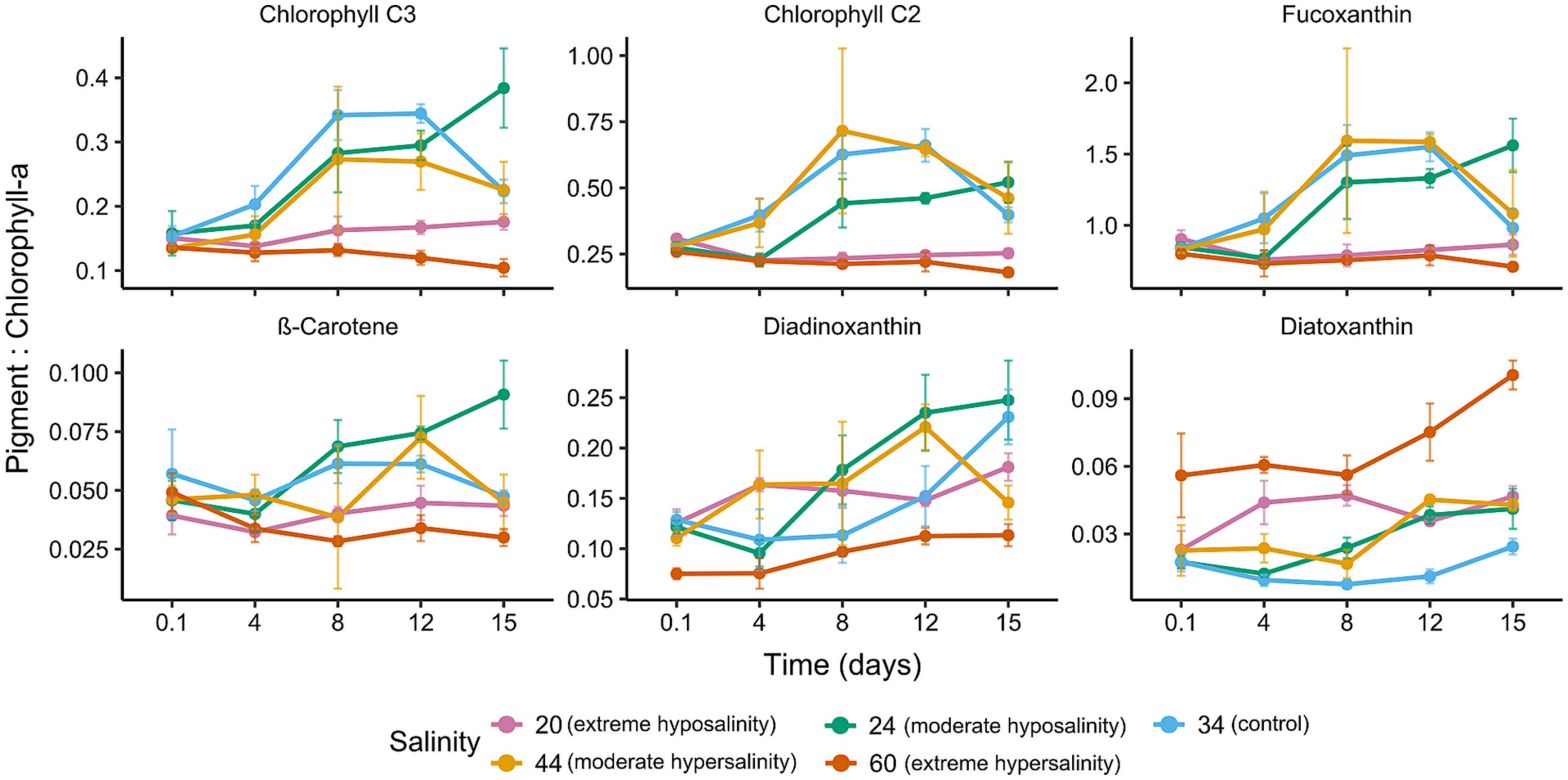
Time series of pigment: Chl *a* ratios for marker pigments (Chl *c*_3_, Chl *c*_2_, fucoxanthin) and photoprotective pigments [*β*-carotene, diadinoxanthin (DD), and diatoxanthin] for each salinity. Lines = mean (*n* = 3), shaded area = ± SD. Panels indicate changes in pigment allocation over time.

HPLC analysis revealed substantial shifts in pigment composition across treatments. Fucoxanthin and chlorophyll c concentrations (Figure 7) were highest in the control and 44‰ cultures. Marker pigment concentrations at 24‰ were comparatively lower but showed a net increase over time. At 20‰ and 60‰, pigment concentrations were significantly reduced.

**Figure 8:**
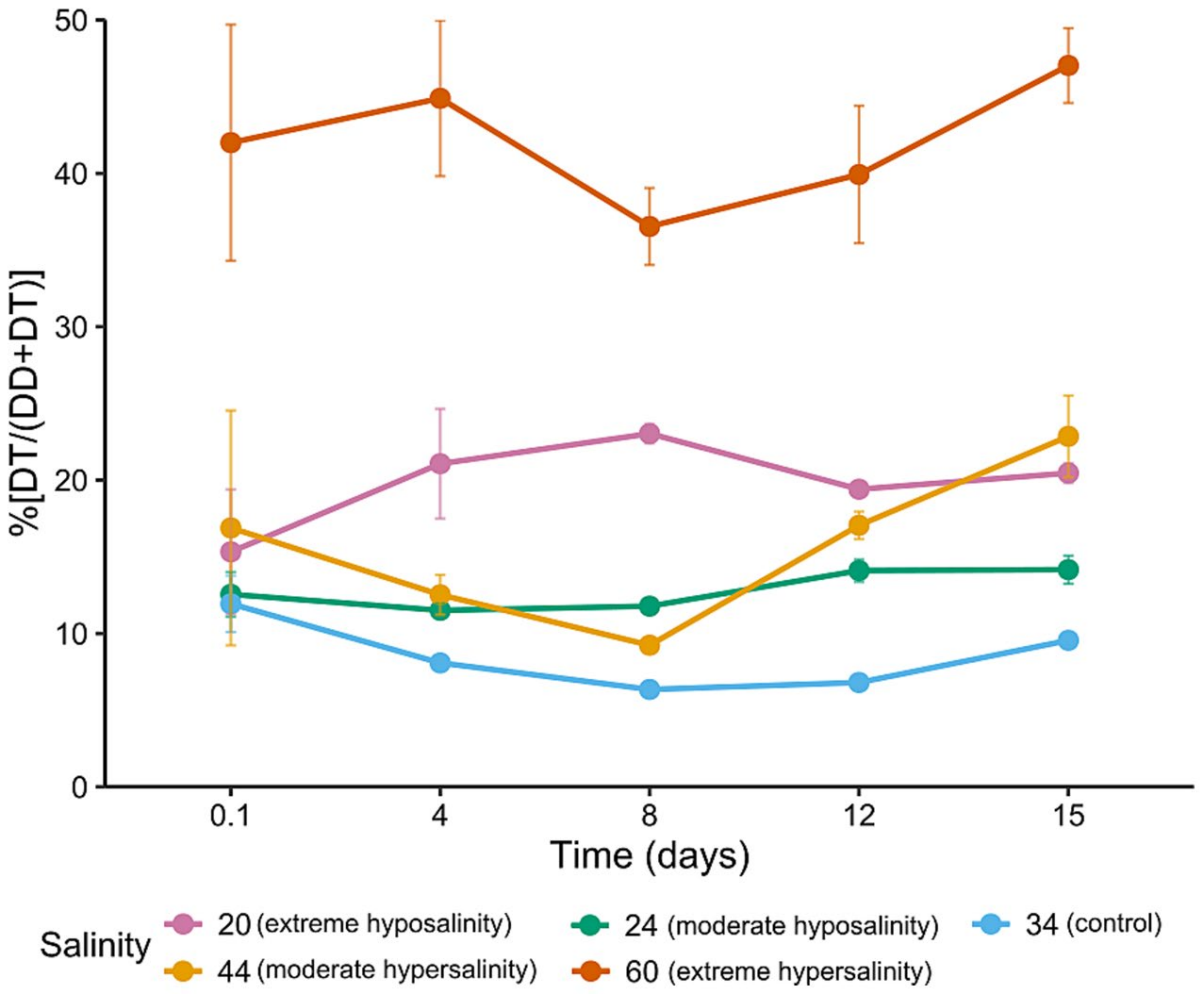
Percent de-epoxidation state (%DES = DT/(DD + DT) × 100) across salinities and time points; error bars = ± SD (*n* = 3).

Considering photoprotective pigments (Figure 7), diatoxanthin (DT) was notably elevated in hypersaline conditions (60‰), indicating activation of non-photochemical quenching (NPQ) and oxidative stress responses. The de-epoxidation state [DT/(DD + DT); Figure 8] increased significantly at 60‰, suggesting sustained engagement of the xanthophyll cycle to mitigate light-induced damage. These pigment dynamics reflect the physiological plasticity of *Chaetoceros* spp., as the wide range of responses—from reduced photosynthetic pigments at low and high salinities to strong upregulation of photoprotective pigments at 60‰ and gradual pigment recovery at 24‰—demonstrates the ability to flexibly adjust different components of the pigment apparatus in response to contrasting salinity-induced pressures.

### 3.5 Nutrient uptake and stoichiometric shifts

**Figure 9:**
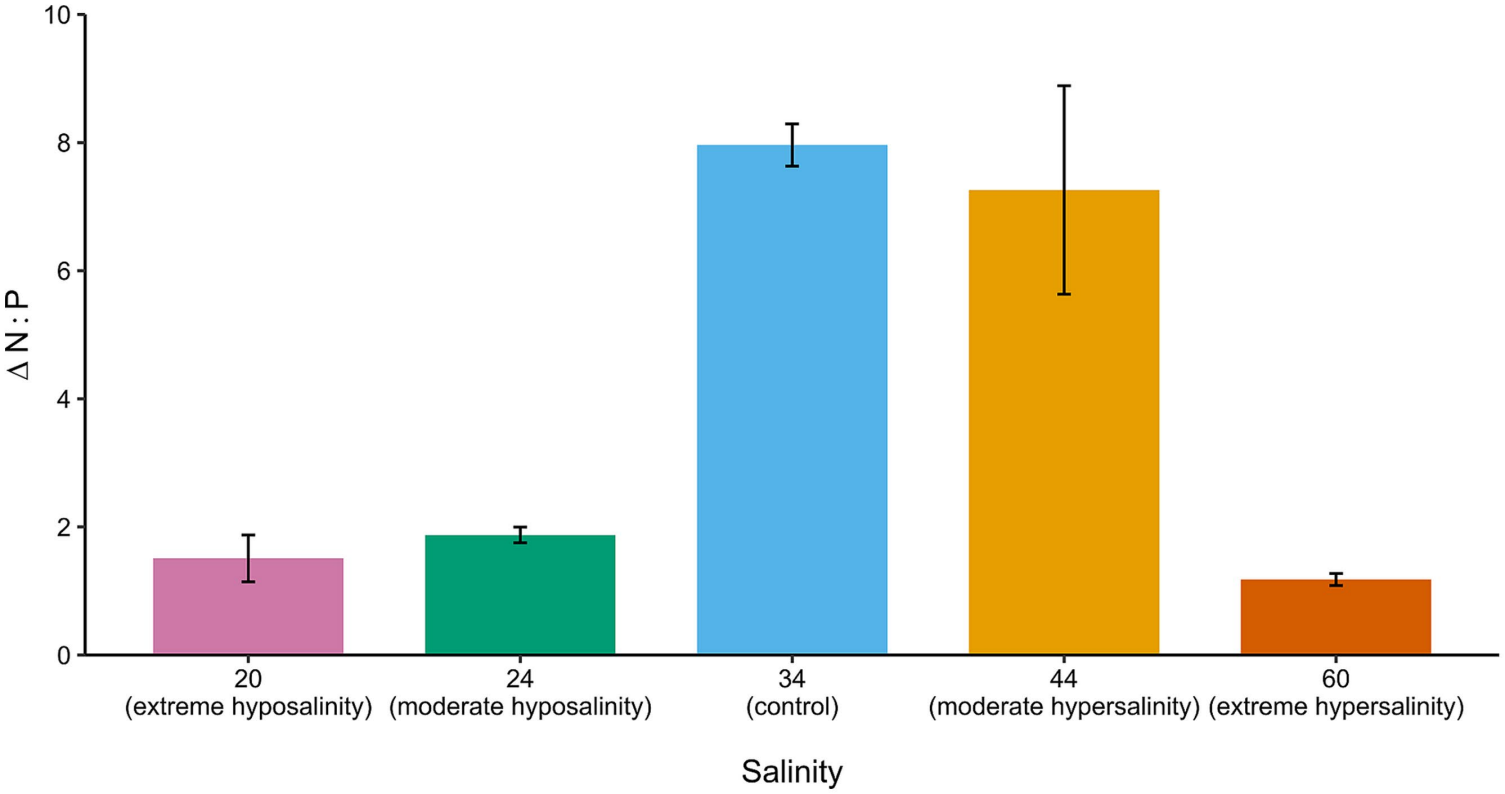
Change in media N:P ratio from day 0.1 (2 h) to day 15 for all salinities. Bars indicate mean fold-change; error bars = ± SD.

Nitrate and phosphate uptake rates (Table 1) varied across treatments, revealing salinity-dependent nutrient assimilation strategies. At 24‰, cultures exhibited impaired nutrient uptake despite high growth rates, resulting in elevated N:P ratios (Table 1; Figure 9). This could signal a potential stoichiometric imbalance.

**Table 1:** Media nutrient concentrations (N-NO_3_^−^ and P-PO_4_^3−^ in μM L^−1^) and N:P ratio on day 0.1 (2 h) and day 15 for each salinity treatment.

Salinity	Day 0.1 (2 h)			Day 15		
	N-NO_3_^−^ (μM/L)	P-PO_4_^3−^ (μM/L)	N:P	N-NO_3_^−^ (μM/L)	P-PO_4_^3−^ (μM/L)	N:P
20 (Extreme hyposalinity)	886.69 ± 14.50	16.84 ± 0.13	52.67 ± 1.24	756.71 ± 56.72	9.74 ± 1.26	79.20 ± 17.35
24 (Moderate hyposalinity)	870.40 ± 68.70	15.66 ± 1.55	60.65 ± 6.16	799.42 ± 31.48	7.06 ± 0.133	113.27 ± 6.32
34 (Control)	885.93 ± 30.77	23.16 ± 0.73	38.25 ± 0.12	759.61 ± 22.95	2.49 ± 0.05	304.56 ± 12.72
44 (Moderate hypersalinity)	824.60 ± 23.17	20.28 ± 0.43	40.65 ± 0.29	731.14 ± 17.67	2.58 ± 0.66	294.83 ± 64.44
60 (Extreme hypersalinity)	943.58 ± 7.86	24.75 ± 1.26	38.18 ± 1.71	904.36 ± 29.84	20.13 ± 0.39	44.93 ± 1.79

Values are mean ± SD (*n* = 3).

Cultures at 44‰ maintained more balanced nutrient uptake, supporting slower but more efficient growth. Extreme salinities disrupted nutrient assimilation: at 20‰, uptake was erratic and inconsistent, while at 60‰, nutrient assimilation was nearly halted. Given the near-complete suppression of nutrient assimilation at 60‰, the short-term growth observed under hypersaline conditions is unlikely to be sustainable over longer timescales.

### 3.6 Principal component analysis

**Figure 10:**
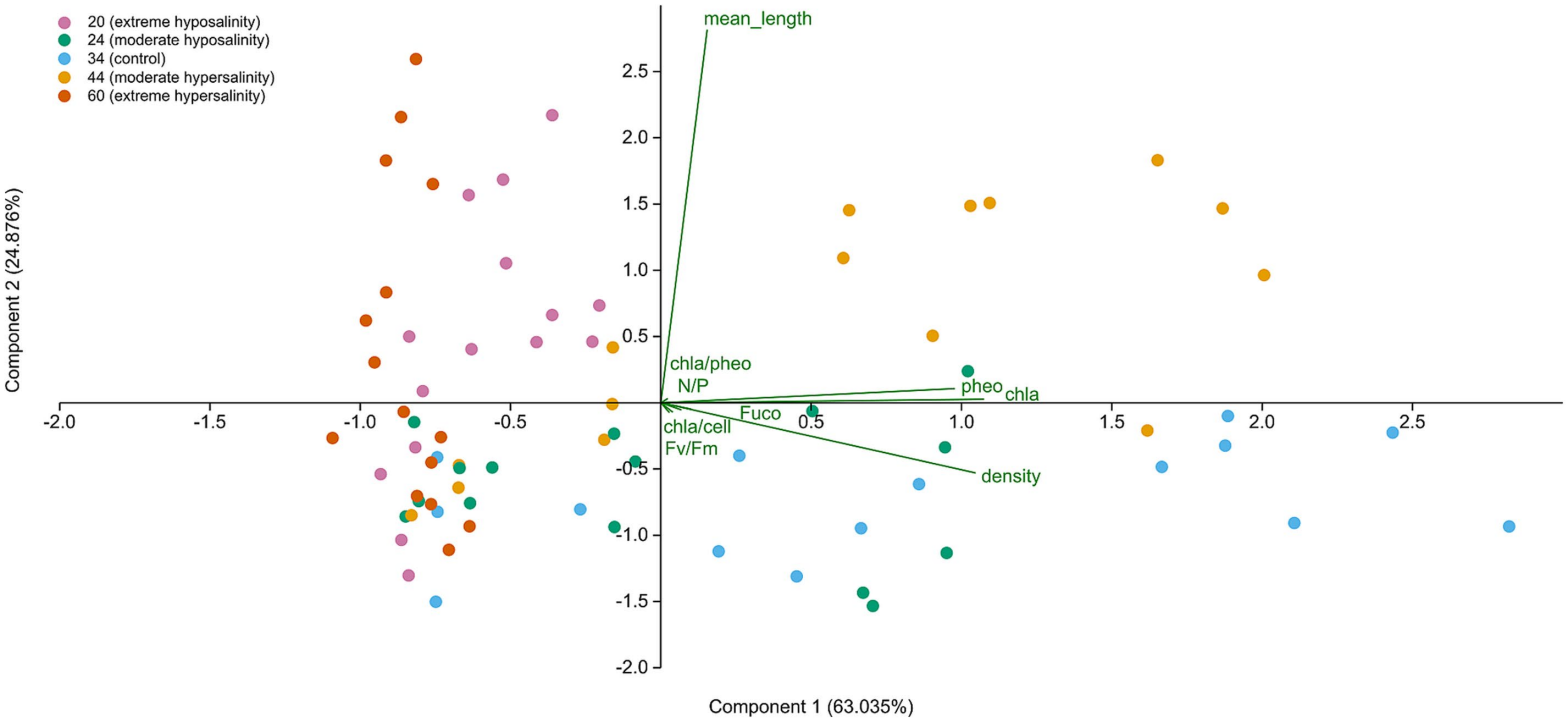
Principal component analysis (PCA) of *Chaetoceros* cultures exposed to different salinity treatments, represented by colored dots.

PCA (Figure 10) revealed clear separation of samples across salinity treatments and time points. PC1 (63.0%) primarily captured variation associated with pigment content, cell density, and Chl a-based traits, while PC2 (24.9%) reflected differences in nutrient stoichiometry and cell morphology. Samples exposed to extreme salinity levels (20‰ and 60‰) clustered distinctly from the control and moderate treatments, indicating divergent multivariate trait profiles. These patterns were statistically supported by a two-way PERMANOVA (Supplementary Table S1; Bray–Curtis index; permutations = 9,999), which detected significant effects of salinity (*F* = 5387.2, *p* = 0.0018) and time (*F* = 6036.6, *p* = 0.0006), with no significant interaction (*F* = −1307.4, *p* = 1).

## 4 Discussion

Responses clustered more strongly by stress severity (moderate vs. extreme) than by the direction (hypo vs. hypersalinity) of salinity change alone. Moderate treatments (24‰ and 44‰) supported growth relative to the extremes but through contrasting physiological adjustments. At 24‰, *Chaetoceros* exhibited reduced cell size and lower pigment content per cell, together with a measurable decline in growth rate. These patterns indicate that moderate hyposalinity imposed physiological stress that constrained both biomass production and pigment investment, rather than promoting a shift toward numerical proliferation. The observed decrease in cell size in 24‰ (with more deformation, lysis, and amorphous cells compared with the control) reflects a regulated adjustment under osmotic stress rather than an increase in division rate. Impaired nutrient uptake at 24‰ further supports the conclusion that hyposalinity reduced overall metabolic efficiency.

At 44‰, cells increased in size and pigment per cell but still exhibited slower population growth than the control. This suggests that moderate hypersalinity induced a different allocation pattern—characterized by greater per-cell investment—yet nevertheless resulted in reduced growth. The modest increase in Fv/Fm at 44‰ fell within the range of variability and did not translate into enhanced growth or nutrient assimilation. Fucoxanthin and chlorophyll c concentrations (Figure 7) were highest in control and 44‰ cultures, consistent with increased pigment investment under moderate hypersalinity.

These results indicate that both moderate hypo- and hypersalinity impaired growth, but the magnitude of the effect was asymmetrical: low salinity (24‰) caused stronger reductions in intact cells, growth rate, and phosphate use than the equivalent increase in salinity (44‰).

Extreme salinities (20‰ and 60‰) exceeded effective tolerance ranges for much of the culture. At 20‰, cells exhibited marked deformation and lysis with only a small surviving fraction showing increased size and partial Fv/Fm recovery, reflecting selection for tolerant subpopulations or osmotic swelling artifacts (Gleitz and Thomas, 1992; Hernando et al., 2015). At 60‰, sustained high %DES and elevated photoprotective pigment levels suggest oxidative and photoprotective stress; persistent photoprotection likely consumed energetic reserves and limited growth capacity, contributing to collapse of chlorophyll pools and population density (Petrou et al., 2011; Kholssi et al., 2023). These observations align with previous findings that *Chaetoceros* spp. tolerate moderate salinity variation but suffer at extremes (Gleitz and Thomas, 1992; Hernando et al., 2018; Liang et al., 2014). Given the near-complete suppression of nutrient assimilation at 60‰, the limited growth observed under hypersaline conditions is unlikely to be sustainable beyond the short term.

Smaller, less pigmented cells promoted by moderate hyposalinity (24‰) reflect reduced per-cell investment and impaired nutrient uptake, consistent with the physiological stress indicated by the decline in growth rate. Increased deformation and lysis under severe hyposalinity (20‰) favor microbial recycling and reduce POC export (Isla et al., 2006). Conversely, larger, more pigmented cells at 44‰ indicate increased per-cell investment under moderate hypersalinity, although a reduced growth rate suggests that this adjustment did not translate into enhanced biomass production.

Pigment:Chl a ratios varied with salinity and time; marker pigments exhibited broader ranges in control and moderate treatments than in extremes. Photoprotective pigments were particularly sensitive to salinity stress (Demers et al., 1991; Lavaud, 2007; Goss and Jakob, 2010; Brunet et al., 2011). The salinity-specific pigment ratios provided in Table 2 can refine CHEMTAX initial matrices for *Chaetoceros*-dominated samples (Neveux and Panouse, 1987; Mackey et al., 1996; Mantoura and Repeta, 1997; Lazzara et al., 2010; Higgins et al., 2011); however, field application requires caution because pigment ratios are influenced by multiple co-occurring environmental factors. Moreover, the pigment responses observed here represent short-term acclimation patterns; their consistency over longer periods is uncertain, as pigment ratios are likely to vary depending on the duration of salinity exposure and the rate at which salinity fluctuates. These patterns indicate that salinity stress alters nutrient demand and uptake efficiency, with implications for cellular stoichiometry, competitive dynamics, and biogeochemical cycling (see Table 3).

**Table 2:** Mean pigment:chl a ratios (Chl c3, Chl c2, fucoxanthin, DD, DT, and β-carotene) for *Chaetoceros* spp. over 15 days (*n* = 15) for each salinity treatment (provided mean ± SD).

Salinity	Chl c3	Chl c2	Fuco	DD	DT	β-Car
20 (Extreme hyposalinity)	0.1588 ± 0.0175	0.2534 ± 0.0333	0.8286 ± 0.0719	0.1551 ± 0.0214	0.0393 ± 0.0109	0.0400 ± 0.0064
24 (Moderate hyposalinity)	0.2579 ± 0.0946	0.3854 ± 0.1265	1.1620 ± 0.3379	0.1756 ± 0.0670	0.0266 ± 0.1247	0.0639 ± 0.0210
34 (Control)	0.2532 ± 0.0824	0.4727 ± 0.1576	1.1822 ± 0.3199	0.1468 ± 0.0512	0.0141 ± 0.0069	0.0546 ± 0.0107
44 (Moderate hypersalinity)	0.2118 ± 0.0775	0.4941 ± 0.2164	1.2130 ± 0.4378	0.1611 ± 0.0468	0.0302 ± 0.0132	0.0500 ± 0.0189
60 (Extreme hypersalinity)	0.1239 ± 0.0146	0.2193 ± 0.0316	0.7574 ± 0.0572	0.0947 ± 0.0199	0.0697 ± 0.0199	0.0351 ± 0.0089

**Table 3:** Summary of key physiological endpoints by salinity treatment: Final density, maximum Fv/Fm, final Chl a, % intact single cells on day 15, and N:P fold-change.

Salinity	Density (cells × L^−1^) day 15	Maximum Fv/Fm	Chl a (μg × L^−1^) day 15	% Intact single cells day 15	N:P fold change
20 (Extreme hyposalinity)	4.592E + 05 ± 1.317E+05	0.423 ± 0.007 (d 8)	16.685 ± 1.738	54.333	1.509 ± 0.366
24 (Moderate hyposalinity)	1.958E + 06 ± 7.001E+04	0.498 ± 0.047 (d 8)	55.245 ± 4.395	83.333	1.875 ± 0.123
34 (Control)	2.264E + 06 ± 1.778E+05	0.531 ± 0.007 (d 8)	102.524 ± 2.950	94.000	7.962 ± 0.340
44 (Moderate hypersalinity)	1.704E + 06 ± 4.219E+05	0.551 ± 0.017 (d 0.1)	90.284 ± 4.741	90.667	7.260 ± 1.628
60 (Extreme hypersalinity)	4.395E + 05 ± 1.632E+05	0.367 ± 0.093 (d 0.1)	3.641 ± 0.225	79.667	1.179 ± 0.093

Preliminary comparison with a co-isolated *Prorocentrum* sp. (exposed to identical hyposaline and hypersaline treatments in a parallel study; unpublished data) demonstrates functional-group specificity: *Prorocentrum* fully recovered at 20‰ by day 15, whereas *Chaetoceros* exhibited partial recovery only; while at 60‰, *Prorocentrum* collapsed entirely, while *Chaetoceros* persisted with impaired function. Such differences suggest that freshening favors some flagellate groups over diatoms under particular regimes, with community-level implications reminiscent of shifts observed on the Western Antarctic Peninsula (Bolinesi et al., 2020a; Mendes et al., 2013, 2018, 2023; Hamilton et al., 2021).

Multivariate patterns indicate that salinity acts as a strong ecological filter shaping phytoplankton functional traits. The consistent clustering of samples along the PCA axes, together with the PERMANOVA outcomes, demonstrates that salinity and time independently structure physiological trajectories. However, the physiological adjustments documented reflect short-term responses to a constant salinity shift; in natural settings, fluctuating or transient salinity changes elicit different or more variable pigment and growth responses.

These results highlight the central role of salinity in modulating trait expression and ecological function in polar phytoplankton communities. This study imposed a single salinity shift and maintained it throughout the exposure period; however, natural polar environments are characterized by rapid, transient brine pulses, and cyclic salinity fluctuations driven by ice formation and melt dynamics (Eicken, 2003; Peterson, 2018). To better simulate *in situ* conditions, future studies must incorporate pulsed and oscillatory salinity regimes, alongside additional environmental drivers such as temperature, irradiance, and nutrient availability (Dong et al., 2016; Hernando et al., 2018). Molecular identification of species within the culture is also essential to resolve intra-genus variability and clarify species-specific responses (Gogorev and Samsonov, 2016; Mock and Junge, 2007). Automated cell enumeration may help reduce observer bias, while direct measurements of reactive oxygen species and osmolyte accumulation may clarify underlying stress mechanisms (Plettner, 2002; Janknegt et al., 2008).

Although species composition within the mixed *Chaetoceros* culture remains to be molecularly resolved, its use reflects the ecological complexity of Antarctic coastal phytoplankton communities, where multiple strains and closely related species co-occur and interact within highly variable microenvironments (Mangoni et al., 2009; Saggiomo et al., 2017; Saggiomo et al., 2021a). Mixed cultures thus offer a more realistic framework for assessing community-level responses to salinity stress, while capturing potential compensatory or synergistic dynamics that may be masked in clonal systems. While species-level attribution is inherently limited, the functional endpoints evaluated—growth dynamics, pigment regulation, and nutrient uptake—are broadly representative of *Chaetoceros*-dominated assemblages (Crosta et al., 1997; Pelusi et al., 2020). The consistent and interpretable patterns observed across various treatments suggest that the responses captured are biologically meaningful and ecologically relevant. Nonetheless, future studies integrating clonal isolates and molecular tools may be critical to disentangle intra- and inter-specific mechanisms and refine trait-based predictions under evolving polar climate scenarios (Hamilton et al., 2021; Mendes et al., 2023; Stuart et al., 2025).

## 5 Conclusion

*Chaetoceros* spp. from Terra Nova Bay exhibited marked physiological plasticity across the salinity gradient, with responses strongly dependent on stress severity. Moderate deviations from ambient salinity supported growth of these diatoms but altered cell size, pigment allocation, and nutrient uptake, while extreme salinities led to structural damage, pigment loss, and impaired population performance. Salinity-driven pigment shifts also affect chemotaxonomic resolution by altering pigment:chlorophyll a ratios used as markers in CHEMTAX, potentially changing the detectability and relative contribution of diatoms and other functional groups in a changing Southern Ocean. The observed acclimation patterns further indicate that salinity acts as an important ecological filter in Antarctic coastal systems.

Within the limits of the measured parameters, our results show that salinity modulates growth, pigment investment, and nutrient assimilation. These physiological adjustments influence cellular resource use and contribute to shaping community responses under climate-driven freshening. Although broader implications for energy transfer and biogeochemical fluxes cannot be directly assessed from this dataset, the contrasting responses observed under moderate vs. extreme salinity shifts suggest potential consequences for ecosystem functioning.

Overall, these findings highlight salinity as a central driver of phytoplankton physiological responses in polar environments, with possible downstream effects on community resilience, trophic interactions, and the functioning of Southern Ocean ecosystems under ongoing climate change.

## Data Availability

The raw data supporting the conclusions of this article will be made available by the authors, without undue reservation.
