# Supplementary material for: Ecological responses of Antarctic Chaetoceros spp. to simulated melting and salinity shifts
Source: Front Microbiol. 2026 Feb 19;17:1750888. doi: 10.3389/fmicb.2026.1750888 (PMC12960622; doi:10.3389/fmicb.2026.1750888)
Supplement: Supplementary file 1 [file Table_1.docx]

**Table S1** – Results of the two-way PERMANOVA and PCA analyses on the considered variables. The table reports the outcomes of the two-way PERMANOVA and the eigenvalues and loadings for all principal components derived from the PCA.

| **Two-way PERMANOVA** |  |  |  |  |  |
| --- | --- | --- | --- | --- | --- |
|  |  |  |  |  |  |
| **Permutation N:** | 9999 |  |  |  |  |
|  |  |  |  |  |  |
| **Source** | **Sum of sqrs** | **df** | **Mean square** | **F** | **p** |
| **sample** | 18088.9 | 4 | 4522.2 | 5387.2 | 0.0018 |
| **time** | 20269.3 | 4 | 5067.3 | 6036.6 | 0.0006 |
| **Interaction** | -17559 | 16 | -1097.4 | -1307.4 | 1 |
| **Residual** | 41.9718 | 50 | 0.83944 |  |  |
| **Total** | 20841 | 74 |  |  |  |

**PRINCIPAL COMPONENT ANALYSE**

| \|  \| **PC 1** \| **PC 2** \| **PC 3** \| **PC 4** \| **PC 5** \| **PC 6** \| **PC 7** \| **PC 8** \| **PC 9** \| \| --- \| --- \| --- \| --- \| --- \| --- \| --- \| --- \| --- \| --- \| \| **chla** \| 0.59625 \| 0.009247 \| -0.33688 \| -0.70536 \| 0.093681 \| 0.13214 \| 0.083354 \| 0.011261 \| 0.007791 \| \| **pheo** \| 0.54257 \| 0.037352 \| 0.82037 \| 0.024978 \| -0.0393 \| -0.15846 \| -0.06259 \| -0.00404 \| 0.001539 \| \| **density** \| 0.58077 \| -0.18454 \| -0.40637 \| 0.65473 \| -0.13521 \| 0.027275 \| -0.12348 \| -0.02301 \| -0.00621 \| \| **chla/pheo** \| 0.012955 \| 0.011248 \| 0.1898 \| 0.13325 \| 0.033147 \| 0.92765 \| 0.28845 \| 0.032971 \| 0.000556 \| \| **chla/cell_pgL** \| 0.015858 \| -0.02593 \| 0.006149 \| 0.080445 \| 0.57704 \| 0.009592 \| -0.12332 \| -0.14232 \| 0.78996 \| \| **Fv/Fm** \| 0.022061 \| -0.02483 \| 0.009406 \| 0.08696 \| 0.76068 \| 0.030711 \| -0.24081 \| 0.082087 \| -0.58902 \| \| **N/P** \| 0.037998 \| -0.01881 \| -0.01923 \| 0.087983 \| 0.12144 \| -0.16795 \| 0.39636 \| 0.8802 \| 0.1236 \| \| **mean_length** \| 0.085488 \| 0.98103 \| -0.10699 \| 0.13546 \| 0.015212 \| -0.00733 \| -0.01278 \| 0.00295 \| 0.005034 \| \| **Fuco** \| 0.056036 \| -0.0168 \| -0.01996 \| 0.12367 \| 0.20907 \| -0.25856 \| 0.81254 \| -0.44326 \| -0.11671 \| |  |  |  |  |  |  |  |  |  |
| --- | --- | --- | --- | --- | --- | --- | --- | --- | --- | --- | --- | --- | --- | --- | --- | --- | --- | --- | --- | --- | --- | --- | --- | --- | --- | --- | --- | --- | --- | --- | --- | --- | --- | --- | --- | --- | --- | --- | --- | --- | --- | --- | --- | --- | --- | --- | --- | --- | --- | --- | --- | --- | --- | --- | --- | --- | --- | --- | --- | --- | --- | --- | --- | --- | --- | --- | --- | --- | --- | --- | --- | --- | --- | --- | --- | --- | --- | --- | --- | --- | --- | --- | --- | --- | --- | --- | --- | --- | --- | --- | --- | --- | --- | --- | --- | --- | --- | --- | --- | --- | --- | --- | --- | --- | --- | --- | --- | --- | --- |
|  |  |  |  |  |  |  |  |  |  |
|  |  |  |  |  |  |  |  |  |  |
| \|  \| \|  \| \|  \| \| \| --- \| --- \| --- \| --- \| --- \| --- \| \|  \|  \| \|  \| \| \| **PC** \| \| **Eigenvalue** \| \| **% variance** \| \| \| **1** \| \| 2.56829 \| \| 63.035 \| \| \| **2** \| \| 1.01352 \| \| 24.876 \| \| \| **3** \| \| 0.359777 \| \| 8.8303 \| \| \| **4** \| \| 0.0915921 \| \| 2.248 \| \| \| **5** \| \| 0.0230494 \| \| 0.56572 \| \| \| **6** \| \| 0.010736 \| \| 0.2635 \| \| \| **7** \| \| 0.00658964 \| \| 0.16173 \| \| \| **8** \| \| 0.000541001 \| \| 0.013278 \| \| \| **9** \| \| 0.000267329 \| \| 0.0065612 \| \| \|  \| \| \|  \| \|  \| \| \|  \|  \| \|  \| \| \|  \|  \| \|  \| \| \|  \|  \| \|  \| \| \|  \|  \| \|  \| \| \|  \|  \| \|  \| \| \|  \|  \| \|  \| \| \|  \|  \| \|  \| \| \|  \|  \| \|  \| \| \|  \|  \| \|  \| \| \|  \| \|  \| \|  \| \| \|  \| \|  \| \|  \| \| \|  \| \|  \| \|  \| \| \|  \| \|  \| \|  \| \| \|  \| \|  \| \|  \| \| \|  \| \|  \| \|  \| \| \|  \| \|  \| \|  \| \| \|  \| \|  \| \|  \| \| \|  \| \|  \| \|  \| \| |  |  |  |  |  |  |  |  |  |
|  |  |  |  |  |  |  |  |  |  |
|  |  |  |  |  |  |  |  |  |  |
|  |  |  |  |  |  |  |  |  |  |
|  |  |  |  |  |  |  |  |  |  |
|  |  |  |  |  |  |  |  |  |  |
|  |  |  |  |  |  |  |  |  |  |
